# (*Z*)-4-[(Ethyl­amino)(furan-2-yl)methyl­idene]-3-methyl-1-phenyl-1*H*-pyrazol-5(4*H*)-one

**DOI:** 10.1107/S1600536812013712

**Published:** 2012-04-04

**Authors:** Li-Nan Li, Wei-Guo Zhang, Shan-Shan Huang, Chuan-Xun Li, Shou-Yu Wang

**Affiliations:** a Department of Orthopaedics, The First Affiliated Hospital of Dalian Medical University, Dalian 116011, People’s Republic of China; b College of Pharmacy, Dalian Medical University, Dalian 116044, People’s Republic of China

## Abstract

In the crystal of the title compound, C_17_H_17_N_3_O_2_, the mol­ecules exist in the keto–enamine form. The pyrazole ring is oriented at 10.59 (4) and 57.98 (5)° to the phenyl and furyl rings, respectively, and the dihedral angle between phenyl and furyl rings is 73.30 (11)°. An intra­molecular N—H⋯O hydrogen bond occurs between imino and carbonyl groups. In the crystal, weak C—H⋯O hydrogen bonds link the mol­ecules into supra­molecular chains along the *b* axis.

## Related literature

For general background to acyl­pyrazolo­nes, see: Dong *et al.* (1983 ▶); Casas *et al.* (2007 ▶). For related structures, see: Zhang *et al.* (2007 ▶); Li *et al.* (2009 ▶); Wang (2010 ▶).
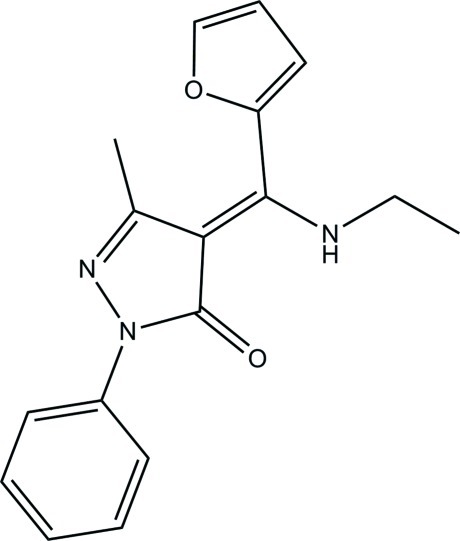


## Experimental

### 

#### Crystal data

C_17_H_17_N_3_O_2_
*M* *_r_* = 295.34
Orthorhombic, 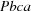

*a* = 8.5729 (13) Å
*b* = 17.555 (3) Å
*c* = 20.427 (3) Å
*V* = 3074.2 (8) Å^3^
*Z* = 8
Mo *K*α radiation
μ = 0.09 mm^−1^
*T* = 296 K
0.20 × 0.18 × 0.16 mm

#### Data collection

Bruker SMART 1000 CCD diffractometer
14892 measured reflections
3552 independent reflections
2271 reflections with *I* > 2σ(*I*)
*R*_int_ = 0.034

#### Refinement

*R*[*F* ^2^ > 2σ(*F* ^2^)] = 0.049
*wR*(*F* ^2^) = 0.153
*S* = 1.02
3552 reflections
202 parameters
H-atom parameters constrained
Δρ_max_ = 0.29 e Å^−3^
Δρ_min_ = −0.24 e Å^−3^

### 

Data collection: *SMART* (Bruker, 1998 ▶); cell refinement: *SAINT* (Bruker, 1998 ▶); data reduction: *SAINT*; program(s) used to solve structure: *SHELXTL* (Sheldrick, 2008 ▶); program(s) used to refine structure: *SHELXTL*; molecular graphics: *SHELXTL*; software used to prepare material for publication: *SHELXTL*.

## Supplementary Material

Crystal structure: contains datablock(s) I, global. DOI: 10.1107/S1600536812013712/xu5499sup1.cif

Structure factors: contains datablock(s) I. DOI: 10.1107/S1600536812013712/xu5499Isup2.hkl

Supplementary material file. DOI: 10.1107/S1600536812013712/xu5499Isup3.cml

Additional supplementary materials:  crystallographic information; 3D view; checkCIF report

## Figures and Tables

**Table 1:** Hydrogen-bond geometry (Å, °)

*D*—H⋯*A*	*D*—H	H⋯*A*	*D*⋯*A*	*D*—H⋯*A*
N3—H3*A*⋯O1	0.98	1.87	2.702 (2)	140
C15—H15⋯O1^i^	0.93	2.39	3.279 (3)	161

Symmetry code: (i) 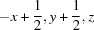.

**Fig. 1.**
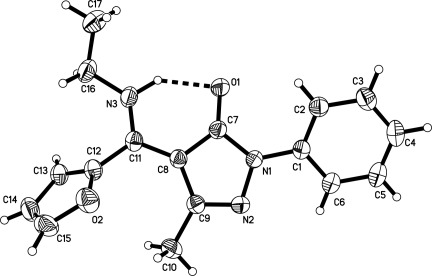
The molecular structure of (I) (thermal ellipsoids are shown at 30% probability levels).

## supplementary crystallographic information

### Comment

1-Phenyl-3-methyl-4-(2-furoyl)-5-pyrazolone (PMFP), is a member of a family of acylpyrazolones, first synthesized in 1983 (Dong *et al.*, 1983). Such acylpyrazolones derivatives form a very important class of heterocycles due to their properties and applications (Casas *et al.*, 2007). We now report the synthesis and structure of the title compound, (I), (Fig. 1).

The molecular structure of (I) is shown in Fig.1. Atoms O1, C7, C8 and C11 of the PMFP moiety and atom N3 of ethylamine group are coplanar, the largest deviation being 0.0517 (11) Å for atom C8. The dihedral angle between this mean plane and pyrazole ring of PMFP is 2.73 (3)°. The bond length of C8—C11 (1.400 (2) Å) between the usual C—C and C=C bonds indicates the delocalization of the electrons because of the addition of a proton to N3 is more favorable than to O2. The atom O2 of PMFP moiety and the N3 atom of ethylamine group are on the same side of C8—C11 bond, which are available for coordination with metal cations. A strong intramolecular hydrogen bond N3—H3A···O1 (Table 1) is also indicative of the enamine-keto form. All bond lengths and angles are normal and comparable with those found in the related compounds (Zhang *et al.*, 2007; Li *et al.*, 2009; Wang, 2010).

### Experimental

A mixture of a 10 ml 1-phenyl-3-methyl-4-(2-furoyl)-5-pyrazolone (2 mmol, 0.5366 g) anhydrous ethanol solution, and a 0.25 ml ethylamine (2 mmol, 0.1306 g) solution was refluxed for *ca* 7 h, with addition of a few drops of glacial acetic acid as a catalyst. The ethanol was removed by evaporation and the resulting green precipitate formed was filtered off, washed with cold anhydrous ethanol and dried in air. Yellow block single crystals suitable for analysis were obtained by slow evaporation of a solution in anhydrous ethanol at room temperature for a few days.

### Refinement

The H3A atom bonded to N3 was located in a difference map and refined in riding mode with *U*_iso_(H) = 1.2*U*_eq_(N). Other H atoms were placed in calculated positions, with C—H = 0.93 Å for phenyl, 0.96 Å for methyl and 0.97 Å for methylene H atoms, and refined as riding, with *U*_iso_(H) = 1.2*U*_eq_(C) for phenyl and methylene H, and 1.5_eq_U(C) for methyl H.

### Crystal data

**Table:**

C_17_H_17_N_3_O_2_	*F*(000) = 1248
*M**_r_* = 295.34	*D*_x_ = 1.276 Mg m^−^^3^
Orthorhombic, *P**b**c**a*	Mo *K*α radiation, λ = 0.71073 Å
Hall symbol: -P 2ac 2ab	Cell parameters from 3420 reflections
*a* = 8.5729 (13) Å	θ = 2.5–24.5°
*b* = 17.555 (3) Å	µ = 0.09 mm^−^^1^
*c* = 20.427 (3) Å	*T* = 296 K
*V* = 3074.2 (8) Å^3^	Block, yellow
*Z* = 8	0.20 × 0.18 × 0.16 mm

### Data collection

**Table:**

Bruker SMART 1000 CCD diffractometer	2271 reflections with *I* > 2σ(*I*)
Radiation source: fine-focus sealed tube	*R*_int_ = 0.034
Graphite monochromator	θ_max_ = 27.6°, θ_min_ = 2.5°
ω scans	*h* = −11→11
14892 measured reflections	*k* = −22→14
3552 independent reflections	*l* = −26→25

### Refinement

**Table:**

Refinement on *F*^2^	Secondary atom site location: difference Fourier map
Least-squares matrix: full	Hydrogen site location: inferred from neighbouring sites
*R*[*F*^2^ > 2σ(*F*^2^)] = 0.049	H-atom parameters constrained
*wR*(*F*^2^) = 0.153	*w* = 1/[σ^2^(*F*_o_^2^) + (0.073*P*)^2^ + 0.6048*P*] where *P* = (*F*_o_^2^ + 2*F*_c_^2^)/3
*S* = 1.02	(Δ/σ)_max_ < 0.001
3552 reflections	Δρ_max_ = 0.29 e Å^−^^3^
202 parameters	Δρ_min_ = −0.24 e Å^−^^3^
0 restraints	Extinction correction: *SHELXTL* (Sheldrick, 2008), Fc^*^=kFc[1+0.001xFc^2^λ^3^/sin(2θ)]^-1/4^
Primary atom site location: structure-invariant direct methods	Extinction coefficient: 0.0061 (11)

### Special details

**Table:**

Geometry. All e.s.d.'s (except the e.s.d. in the dihedral angle between two l.s. planes) are estimated using the full covariance matrix. The cell e.s.d.'s are taken into account individually in the estimation of e.s.d.'s in distances, angles and torsion angles; correlations between e.s.d.'s in cell parameters are only used when they are defined by crystal symmetry. An approximate (isotropic) treatment of cell e.s.d.'s is used for estimating e.s.d.'s involving l.s. planes.
Refinement. Refinement of *F*^2^ against ALL reflections. The weighted *R*-factor *wR* and goodness of fit *S* are based on *F*^2^, conventional *R*-factors *R* are based on *F*, with *F* set to zero for negative *F*^2^. The threshold expression of *F*^2^ > σ(*F*^2^) is used only for calculating *R*-factors(gt) *etc*. and is not relevant to the choice of reflections for refinement. *R*-factors based on *F*^2^ are statistically about twice as large as those based on *F*, and *R*- factors based on ALL data will be even larger.

### Fractional atomic coordinates and isotropic or equivalent isotropic displacement parameters (Å^2^)

**Table:**

	*x*	*y*	*z*	*U*_iso_*/*U*_eq_	
C1	0.3274 (2)	0.36448 (10)	0.37402 (8)	0.0470 (4)	
C2	0.4490 (2)	0.31933 (11)	0.35231 (9)	0.0568 (5)	
H2	0.4819	0.3221	0.3090	0.068*	
C3	0.5211 (2)	0.27020 (12)	0.39536 (10)	0.0673 (6)	
H3	0.6030	0.2399	0.3807	0.081*	
C4	0.4740 (3)	0.26520 (13)	0.45952 (11)	0.0719 (6)	
H4	0.5234	0.2317	0.4881	0.086*	
C5	0.3540 (2)	0.30984 (13)	0.48091 (10)	0.0676 (6)	
H5	0.3219	0.3067	0.5243	0.081*	
C6	0.2797 (2)	0.35970 (12)	0.43881 (9)	0.0561 (5)	
H6	0.1981	0.3899	0.4538	0.067*	
C7	0.2554 (2)	0.42127 (10)	0.26479 (8)	0.0500 (4)	
C8	0.1713 (2)	0.48955 (10)	0.24974 (8)	0.0479 (4)	
C9	0.1213 (2)	0.51919 (11)	0.31153 (9)	0.0502 (4)	
C10	0.0283 (3)	0.58819 (12)	0.32686 (10)	0.0672 (6)	
H10A	−0.0075	0.5856	0.3713	0.101*	
H10B	−0.0597	0.5909	0.2979	0.101*	
H10C	0.0921	0.6327	0.3213	0.101*	
C11	0.1465 (2)	0.51345 (10)	0.18519 (9)	0.0506 (4)	
C12	0.0770 (2)	0.58668 (10)	0.16927 (9)	0.0526 (5)	
C13	−0.0461 (2)	0.60530 (12)	0.13197 (9)	0.0561 (5)	
H13	−0.1068	0.5722	0.1072	0.067*	
C14	−0.0655 (3)	0.68398 (15)	0.13746 (12)	0.0791 (7)	
H14	−0.1426	0.7132	0.1175	0.095*	
C15	0.0474 (3)	0.70936 (13)	0.17668 (12)	0.0834 (7)	
H15	0.0622	0.7600	0.1884	0.100*	
C16	0.1841 (3)	0.48181 (15)	0.06678 (10)	0.0774 (7)	
H16A	0.2017	0.5355	0.0585	0.093*	
H16B	0.0818	0.4686	0.0500	0.093*	
C17	0.3046 (3)	0.43617 (17)	0.03265 (11)	0.0931 (8)	
H17A	0.4054	0.4478	0.0506	0.140*	
H17B	0.3036	0.4482	−0.0132	0.140*	
H17C	0.2829	0.3829	0.0385	0.140*	
N1	0.25453 (19)	0.41740 (8)	0.33228 (6)	0.0506 (4)	
N2	0.16849 (17)	0.47679 (9)	0.36000 (7)	0.0530 (4)	
N3	0.1892 (2)	0.46750 (9)	0.13692 (7)	0.0637 (5)	
H3A	0.2364	0.4192	0.1514	0.076*	
O1	0.31504 (18)	0.37337 (8)	0.22731 (6)	0.0658 (4)	
O2	0.13911 (19)	0.64946 (9)	0.19731 (8)	0.0768 (5)	

### Atomic displacement parameters (Å^2^)

**Table:**

	*U*^11^	*U*^22^	*U*^33^	*U*^12^	*U*^13^	*U*^23^
C1	0.0528 (9)	0.0412 (9)	0.0472 (9)	−0.0088 (8)	−0.0069 (7)	0.0016 (7)
C2	0.0628 (11)	0.0525 (11)	0.0552 (10)	−0.0007 (9)	−0.0040 (9)	−0.0025 (8)
C3	0.0633 (12)	0.0594 (13)	0.0793 (14)	0.0061 (10)	−0.0146 (11)	−0.0005 (11)
C4	0.0719 (13)	0.0688 (14)	0.0752 (14)	−0.0025 (12)	−0.0224 (11)	0.0189 (11)
C5	0.0685 (13)	0.0798 (15)	0.0546 (11)	−0.0114 (12)	−0.0103 (9)	0.0163 (11)
C6	0.0566 (10)	0.0607 (12)	0.0511 (10)	−0.0051 (9)	−0.0034 (8)	0.0052 (9)
C7	0.0609 (10)	0.0444 (10)	0.0446 (9)	−0.0025 (9)	−0.0018 (8)	−0.0005 (7)
C8	0.0544 (10)	0.0414 (9)	0.0477 (9)	−0.0033 (8)	−0.0040 (7)	0.0015 (8)
C9	0.0505 (9)	0.0460 (10)	0.0543 (10)	−0.0028 (8)	−0.0012 (8)	0.0005 (8)
C10	0.0674 (12)	0.0613 (13)	0.0728 (13)	0.0131 (11)	0.0052 (10)	−0.0014 (10)
C11	0.0555 (10)	0.0440 (10)	0.0523 (10)	−0.0102 (8)	−0.0052 (8)	0.0043 (8)
C12	0.0509 (10)	0.0480 (11)	0.0589 (11)	−0.0072 (8)	−0.0006 (8)	0.0074 (8)
C13	0.0508 (10)	0.0590 (12)	0.0583 (10)	−0.0014 (9)	−0.0078 (8)	0.0080 (9)
C14	0.0757 (14)	0.0754 (16)	0.0862 (16)	0.0254 (13)	0.0148 (13)	0.0283 (13)
C15	0.108 (2)	0.0462 (13)	0.0959 (17)	−0.0016 (13)	0.0296 (16)	0.0002 (12)
C16	0.1008 (17)	0.0840 (17)	0.0474 (11)	0.0017 (14)	−0.0064 (11)	0.0079 (11)
C17	0.119 (2)	0.101 (2)	0.0587 (13)	0.0073 (17)	0.0081 (13)	−0.0057 (13)
N1	0.0654 (9)	0.0447 (9)	0.0416 (7)	0.0042 (7)	−0.0013 (7)	0.0002 (6)
N2	0.0606 (9)	0.0494 (9)	0.0490 (8)	0.0035 (7)	0.0022 (7)	−0.0035 (7)
N3	0.0953 (13)	0.0510 (10)	0.0447 (8)	0.0010 (9)	−0.0074 (8)	0.0033 (7)
O1	0.0989 (11)	0.0512 (8)	0.0471 (7)	0.0146 (7)	−0.0001 (7)	−0.0042 (6)
O2	0.0834 (10)	0.0573 (9)	0.0896 (11)	−0.0112 (8)	−0.0091 (8)	0.0004 (8)

### Geometric parameters (Å, º)

**Table:**

C1—C2	1.382 (3)	C10—H10C	0.9600
C1—C6	1.388 (3)	C11—N3	1.325 (2)
C1—N1	1.407 (2)	C11—C12	1.454 (3)
C2—C3	1.378 (3)	C12—C13	1.342 (2)
C2—H2	0.9300	C12—O2	1.352 (2)
C3—C4	1.374 (3)	C13—C14	1.396 (3)
C3—H3	0.9300	C13—H13	0.9300
C4—C5	1.365 (3)	C14—C15	1.333 (4)
C4—H4	0.9300	C14—H14	0.9300
C5—C6	1.382 (3)	C15—O2	1.379 (3)
C5—H5	0.9300	C15—H15	0.9300
C6—H6	0.9300	C16—N3	1.455 (2)
C7—O1	1.247 (2)	C16—C17	1.482 (3)
C7—N1	1.380 (2)	C16—H16A	0.9700
C7—C8	1.432 (3)	C16—H16B	0.9700
C8—C11	1.400 (2)	C17—H17A	0.9600
C8—C9	1.431 (2)	C17—H17B	0.9600
C9—N2	1.303 (2)	C17—H17C	0.9600
C9—C10	1.483 (3)	N1—N2	1.397 (2)
C10—H10A	0.9600	N3—H3A	0.9845
C10—H10B	0.9600		
			
C2—C1—C6	119.58 (17)	N3—C11—C12	119.01 (16)
C2—C1—N1	121.26 (16)	C8—C11—C12	122.55 (17)
C6—C1—N1	119.12 (16)	C13—C12—O2	110.60 (17)
C3—C2—C1	119.48 (18)	C13—C12—C11	131.66 (18)
C3—C2—H2	120.3	O2—C12—C11	117.69 (16)
C1—C2—H2	120.3	C12—C13—C14	106.80 (19)
C4—C3—C2	121.1 (2)	C12—C13—H13	126.6
C4—C3—H3	119.4	C14—C13—H13	126.6
C2—C3—H3	119.4	C15—C14—C13	107.0 (2)
C5—C4—C3	119.3 (2)	C15—C14—H14	126.5
C5—C4—H4	120.3	C13—C14—H14	126.5
C3—C4—H4	120.3	C14—C15—O2	110.0 (2)
C4—C5—C6	120.8 (2)	C14—C15—H15	125.0
C4—C5—H5	119.6	O2—C15—H15	125.0
C6—C5—H5	119.6	N3—C16—C17	110.42 (19)
C5—C6—C1	119.7 (2)	N3—C16—H16A	109.6
C5—C6—H6	120.2	C17—C16—H16A	109.6
C1—C6—H6	120.2	N3—C16—H16B	109.6
O1—C7—N1	125.63 (17)	C17—C16—H16B	109.6
O1—C7—C8	129.72 (16)	H16A—C16—H16B	108.1
N1—C7—C8	104.63 (15)	C16—C17—H17A	109.5
C11—C8—C9	132.56 (17)	C16—C17—H17B	109.5
C11—C8—C7	121.97 (16)	H17A—C17—H17B	109.5
C9—C8—C7	105.42 (15)	C16—C17—H17C	109.5
N2—C9—C8	111.69 (16)	H17A—C17—H17C	109.5
N2—C9—C10	118.21 (16)	H17B—C17—H17C	109.5
C8—C9—C10	130.09 (17)	C7—N1—N2	111.78 (14)
C9—C10—H10A	109.5	C7—N1—C1	129.42 (15)
C9—C10—H10B	109.5	N2—N1—C1	118.80 (13)
H10A—C10—H10B	109.5	C9—N2—N1	106.40 (14)
C9—C10—H10C	109.5	C11—N3—C16	128.24 (18)
H10A—C10—H10C	109.5	C11—N3—H3A	114.4
H10B—C10—H10C	109.5	C16—N3—H3A	117.2
N3—C11—C8	118.44 (17)	C12—O2—C15	105.52 (17)
			
C6—C1—C2—C3	0.0 (3)	C8—C11—C12—O2	51.5 (2)
N1—C1—C2—C3	177.63 (17)	O2—C12—C13—C14	−1.2 (2)
C1—C2—C3—C4	0.1 (3)	C11—C12—C13—C14	176.4 (2)
C2—C3—C4—C5	−0.2 (3)	C12—C13—C14—C15	1.0 (2)
C3—C4—C5—C6	0.1 (3)	C13—C14—C15—O2	−0.5 (3)
C4—C5—C6—C1	0.0 (3)	O1—C7—N1—N2	−175.51 (18)
C2—C1—C6—C5	−0.1 (3)	C8—C7—N1—N2	3.0 (2)
N1—C1—C6—C5	−177.75 (17)	O1—C7—N1—C1	5.4 (3)
O1—C7—C8—C11	−1.4 (3)	C8—C7—N1—C1	−176.05 (17)
N1—C7—C8—C11	−179.86 (16)	C2—C1—N1—C7	18.6 (3)
O1—C7—C8—C9	176.14 (19)	C6—C1—N1—C7	−163.80 (18)
N1—C7—C8—C9	−2.32 (19)	C2—C1—N1—N2	−160.46 (16)
C11—C8—C9—N2	178.07 (19)	C6—C1—N1—N2	17.2 (2)
C7—C8—C9—N2	0.9 (2)	C8—C9—N2—N1	0.9 (2)
C11—C8—C9—C10	−2.1 (3)	C10—C9—N2—N1	−178.91 (15)
C7—C8—C9—C10	−179.30 (19)	C7—N1—N2—C9	−2.5 (2)
C9—C8—C11—N3	−170.12 (19)	C1—N1—N2—C9	176.65 (15)
C7—C8—C11—N3	6.7 (3)	C8—C11—N3—C16	−175.9 (2)
C9—C8—C11—C12	10.3 (3)	C12—C11—N3—C16	3.7 (3)
C7—C8—C11—C12	−172.87 (17)	C17—C16—N3—C11	153.0 (2)
N3—C11—C12—C13	54.5 (3)	C13—C12—O2—C15	0.9 (2)
C8—C11—C12—C13	−125.9 (2)	C11—C12—O2—C15	−177.06 (17)
N3—C11—C12—O2	−128.04 (19)	C14—C15—O2—C12	−0.2 (2)

### Hydrogen-bond geometry (Å, º)

**Table:**

*D*—H···*A*	*D*—H	H···*A*	*D*···*A*	*D*—H···*A*
N3—H3*A*···O1	0.98	1.87	2.702 (2)	140
C15—H15···O1^i^	0.93	2.39	3.279 (3)	161

Symmetry code: (i) −*x*+1/2, *y*+1/2, *z*.
